# Longitudinal results of strengthening the parent-team alliance in child semi-residential psychiatry: does team investment make a difference?

**DOI:** 10.1186/s13034-016-0108-5

**Published:** 2016-07-18

**Authors:** Audri Lamers, Chijs van Nieuwenhuizen, Jos Twisk, Erica de Koning, Robert Vermeiren

**Affiliations:** Curium-LUMC, Centre of Child and Youth Psychiatry, Leiden University, Endegeesterstraatweg 27, 2342 Oegstgeest, The Netherlands; GGzE Centre for Child and Adolescent Psychiatry, PO BOX 909 (DP 8001), 5600 Eindhoven, The Netherlands; Tranzo, Scientific Centre for Care and Welfare, Tilburg University, PO BOX 90153, 5000 Tilburg, The Netherlands; Department of Clinical Epidemiology and Biostatistics, Vrije Universiteit Medical Centre, De Boelelaan 1118, 1081 Amsterdam, The Netherlands

**Keywords:** Parents, Therapeutic alliance, Residential psychiatry, Children

## Abstract

**Background:**

In a semi-residential setting where children switch daily between treatment and home, establishment of a strong parent-team alliance can be a challenge. The development of alliance with parents and the symptoms of the child might be strengthened by a structured investment of treatment team members.

**Methods:**

Participants were caregivers and treatment team members of 46 children (6–12 years) who received semi-residential psychiatric treatment. An A–B design was applied, in which the first 22 children were assigned to the comparison group receiving treatment as usual and the next 24 to the experimental group, where treatment team members used additional alliance-building strategies. Alliance and symptom questionnaires were filled out at three-month intervals during both treatment conditions. Parent-treatment team interactions, assessed on DVD, were coded according to members’ adherence to these strategies.

**Results:**

Multilevel analyses (using MLwiN) showed that based on reports of primary caregivers and a case manager, the alliance-building strategies had a statistically significant effect on the strength of the therapeutic alliance between treatment team members and parents. In addition, primary caregivers in the experimental group reported significant less hyperactivity symptoms of their children.

**Conclusions:**

Despite the methodological challenge of examining therapeutic processes in this complex treatment setting, this study supports the benefits of structured investment in the parent-team alliance.

**Electronic supplementary material:**

The online version of this article (doi:10.1186/s13034-016-0108-5) contains supplementary material, which is available to authorized users.

## Background

The therapeutic alliance between therapists and parents is increasingly acknowledged as a key component of the therapeutic process with children and adolescents (hereafter, referred to as *youth*). Commonly, therapeutic alliance is defined as the affective and collaborative aspects of the individual client-therapist relationship [1]. In youth mental health care, however, at least two therapeutic alliances are vigorous: the youth-therapist alliance and the parent-therapist alliance [2]. Interestingly, therapeutic alliances with parents of youth are associated with a wider range of positive outcomes than youth alliances only [3–5]. Parent alliance has been related to youths’ symptom improvements [3, 6, 7], parenting skills improvement [6, 8], more treatment attendance and retention [6, 9], longer term youth adjustment after treatment [4], and more parent satisfaction with therapy [7]. In family therapy the parent alliance has even been identified as a moderator of the relationship between youth’s alliance and treatment outcome [10]. Clearly, the therapeutic alliance of therapists with parents deserves ample attention while improving treatments for youths.

Insufficient empirical evidence exists, until now, to guide therapists in the formation of therapeutic alliances with parents [11]. This is in contrast to adult psychotherapy research that showed the effectiveness of enhancing the client-therapist therapeutic alliance through the training of clinicians [12, 13]. For instance, brief or subtle strategies, such as encouraging clients to give feedback about aspects of the therapeutic process, produced strong and lasting benefits for the therapeutic alliance. Youth psychotherapy research also showed alliance-building behaviors of therapists are associated with stronger growth in the youth-therapist alliance [14–17]. For example, “collaboration” positively influences the youth alliance and “pushing the child to talk” influences it negatively [14]. In a recent meta-analysis of the therapeutic alliance in the youth field, McLeod [11] advocated investigation of factors that influence parent alliance formation and development. While there has been attention for youth and adult alliance building in psychotherapy, the literature on parent alliance building is primarily descriptive [18, 19].

Investment in a strong therapeutic alliance with parents might be especially challenging in a semi-residential setting where youth switch on a daily basis between the treatment setting and home. Due to the high costs and impact of (semi) residential psychiatric treatment in youth mental health care, refinement of effective strategies is a necessity. The importance of the therapeutic alliance with parents in (semi) residential settings is reflected in ample literature describing (a) the dynamics of the parent-treatment team alliance [20], (b) the perspectives of parents and treatment team members on their alliance [2, 21], and (c) ways to positively influence the strength of the parent-treatment team alliance [22, 23]. The parent-treatment team alliance has been identified as a critical component in relation to treatment success for youths in the (semi) residential setting [4, 24]. To elaborate on this research, several authors recommend investigating how the quality of the therapeutic alliance changes over time from different perspectives [2, 25, 26]. Furthermore, as the parent-team therapeutic alliance is posited to be crucial in promoting the outcomes of residential psychiatry, research is needed to the effect of strengthening the parent-team alliance in residential settings.

Therefore, the main objective of this study is to evaluate strengthening of the parent-treatment team therapeutic alliance in a youth semi-residential setting from different perspectives. Alliance building strategies which were delineated from the alliance literature were added to an already existing psychiatric semi-residential intervention for children. Given the previous findings on strengthening effects in the adult alliance during psychotherapy, we hypothesized that the development of alliance with parents can be strengthened by a structured investment of treatment team members in semi-residential psychiatry. In addition, we hypothesized that the child’s symptoms would improve faster during treatment when treatment team members would invest in the therapeutic alliance with parents.

## Methods

### Design

**Fig. 1:**
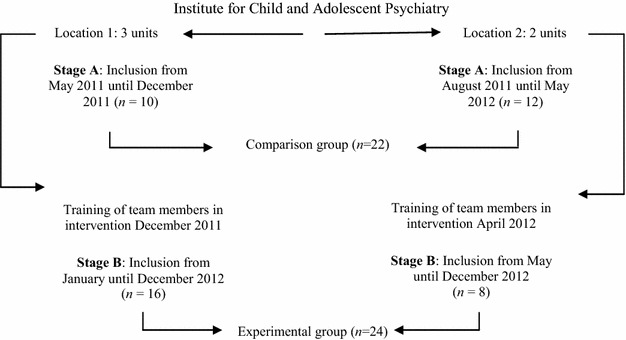
Study design and children’s allocation to groups

This is a longitudinal study using an A–B design implemented at five semi-residential units in two locations of the Institute for Child and Adolescent Psychiatry in the Netherlands. In the first stage (A), the comparison group (*n* = 22) of newly admitted children and their parents received treatment as usual. In the next stage (B), for the experimental group (*n* = 24), team members were trained in alliance-building strategies and applied these with parents and their children in addition to carrying out treatment as usual. A specific treatment manual was developed as well as a structured training protocol, which integrates attention for treatment integrity procedures. Although a randomized controlled trial is preferred for effectiveness research, mutual influencing effects were expected between the comparison and experimental groups. Figure 1 illustrates the allocation of children to a comparison group and experimental group. Inclusion lasted until December 2012.

### Participants

Participants in this study were 46 primary caregivers, two licensed clinical psychologists and eight group workers. The group workers provide a daily structured therapeutic treatment program. At each location, one licensed clinical psychologist is involved as a case manager overall responsible for the children’s diagnostics and treatment and as the coordinator of the whole multidisciplinary team. The children of the caregivers had a mean age of 8.9 (*SD* = 1.6; range 6–12 years). The primary caregiver was the mother; only in one case the primary caregiver was the father. Children attended semi-residential treatment for at least three, but usually five, days a week for 8 h a day (mean days in treatment = 322; *SD* = 116). Characteristics of children and their parents of both treatment groups are presented in Table 1.

**Table 1:** Baseline characteristics of the 46 children and their primary caregiver between treatment conditions

Participants baseline characteristics	Comparison group (*n* = 22)	Experimental group (*n* = 24)	*P*
Age child (mean, *SD*)	8.6	9.2	.24
Days in treatment child (means, *SD*)	328 (102)	248 (123)	*.04*
Girls	18	21	.82
Family composition			
Biological parents	73	67	.70
Single parents	13.5	25	
Other	13.5	8	
Caregiver education level			
Early/primary/secondary	77	79	.66
Bachelor/master/doctoral	23	17	
Missing	4	4	
DSM-IV AXIS I classification child			
PDD	72.7	66.7	*.04*
ADHD/ODD	–	12.5	
Mood and anxiety disorders	18.2	–	
Other disorders	9.1	20.8	
Presence comorbidity on AXIS I	40.9	50	.54

Values given are percentages, unless otherwise indicated

*p* ≤ .05 (italiced)

*PDD* pervasive development disorder; *ADHD/ODD* attention deficit/hyperactivity disorder/oppositional defiant disorder

### Comparison condition

At each location, a multidisciplinary team provided treatment to eight children per unit, which consisted of a therapeutic milieu on the ward, parent counseling/training, educative therapy, psychomotor therapy, and creative therapy. Children were involved in a highly structured day schedule in which social activities and school were integrated. The treatment team consisted of group care workers, parent counselors, a licensed clinical psychologist and if indicated the child psychiatrist, creative, educative, and psychomotor therapists. The primary goal of (semi) residential treatment is to reduce psychiatric symptoms and improve youths’ quality of life and well-being.

### Experimental condition

Based on the literature regarding therapeutic alliance building, therapeutic strategies on a practical level and on a therapeutic level were added to the regular semi-residential treatment to strengthen the parent-team alliance.

#### Practical level

Special alliance-building opportunities were incorporated in the child semi-residential treatment. These alliance opportunities entailed:

- Framework meeting. After intake a pre-treatment meeting takes place in which parents, parent counselor, and case manager mutually design and agree upon a detailed treatment contract.
- Treatment evaluation. Every 3 months during treatment, the treatment plan is evaluated by parents and treatment team and new goals are agreed upon.
- Consent meeting. Every 3 months after intake or evaluation, parents express their consent for the treatment by communicating to their child, in the presence of the treatment team, the goals that have been attained and the rationale for the new goals. All participants sign the treatment plan, creating a ritual that emphasizes the collaboration between parents, child, and treatment team.

#### Therapeutic level

During the whole treatment, and especially in these alliance-building opportunities, the treatment team applied the following therapeutic strategies.

- Partnership. The treatment team strives to obtain a shared vision on diagnose, treatment goals, and tasks, while designing a mutual treatment plan in partnership with parents. The team members frequently emphasize the concept of partnership, mutual collaboration, joint effort, being part of the team and input being of equal importance for the treatment program. When parents are regarded as partners they will invest more intensively and effectively in the treatment program [27, 28]. Partnership strengthens the alliance with parents especially in a (semi) residential setting [20]. Parents are incited by asking to reflect on the child’s development and the treatment policy. In partnerships, when there is equality in decision making, responsibility, and accountability, parents will feel more secure about the agreed upon treatment plans and will express differing opinions early in the course of treatment. Next, parents are in charge of communicating the treatment plan to the child. Research has showed reduced numbers of dropout when children are extensively prepared about the treatment content [29].
- Positive attributions of team members towards parents and positive mutual expectations. Ackerman and Hilsenroth [30] showed in their overview that positive attributions and expectations of clinicians regarding the collaboration with the client, significantly relates to the development and maintenance of a strong therapeutic alliance. Thus, team members should strongly focus on the strengths and competencies of children and parents and their capability to change. When the treatment has a positive effect due to the influence of parents, this is punctuated. In residential treatment, Scharer [23] pleads to explicitly explore expectations of parents and clinicians before admitting a child as these expectations have an influence on the alliance during treatment. Therefore, during the child’s admission process, parents’ expectations and hope for change are explored and reframed as more positive ones.
- Explicitly evaluating the parent-team alliance. In the framework and evaluation meetings, all participants give a scale score between 1 and 10 with regard to the strength of the parent-team alliance. Questions like “How did we succeed in having this score on the scale?” and “What is needed from participants to move the score one point more in the right direction,” are used to move the alliance in a positive direction. When feedback in adult psychotherapy is given about the therapeutic alliance, clients are more likely to experience a clinically significant change [31]. Due to more detailed information about the alliance, team members can adjust their therapeutic attitude or skills.

### Treatment manual, training protocol and integrity procedures

To derive alliance strengthening strategies from the literature, a keyword search was conducted around therapeutic alliance building and collaboration with parents in a (semi-) residential setting. Based on this literature search and the experience in several child semi-residential settings in The Netherlands the optimal parent-team strengthening strategies were described. In collaboration with the involved teams was explored which and how these strategies could be fitted or integrated in the care as usual of the semi-residential settings of Curium-LUMC. The outcome of these brainstorm sessions, which is the strategies described in the former section, was manualized and subsequently reviewed by the teams. Some aspects of alliance strengthening strategies formulated as optimal, such as regular attendance of parents at the unit, were at that moment seen as infeasible.

Integrity of the use and competence of the alliance strategies by team members was evaluated using Perepletchikova’s [32] procedures, which comprise six steps. First, a more specific manual was developed consisting of descriptions of the core therapeutic strategies, the rationales for adherence, and spelling out verbatim statements. Second, team members were trained in these strategies with a step by step training protocol consisting of theoretical background, example DVDs, and practical role-play. Third, meetings were held about once every month, where team members went through the procedures, conducted skype sessions between disciplines, and talked about specific cases. Fourth, the evaluation meeting of the team together with parents, which took place every 3 months, was taped on video. Prior to these meetings, parents were asked for their permission to tape the meeting for this research goal. Fifth, a coding manual was developed to assess adherence to the alliance-building strategies. Eight aspects were rated on a 4-point scale where 1 reflected *no adherence* and 4 reflected *clear adherence*. Sixth, for interrater agreement, 50 % of the recorded DVD’s were scored by a second independent rater.

### Measures

#### Parent-team alliance from team’ perspective

The Dutch Family Engagement Questionnaire (FEQ) is a 14-item questionnaire aimed at assessing the youth and parent therapeutic alliance with team members in the specific setting of child and adolescent psychiatry from the treatment team’s perspective [2]. The FEQ was originally developed in the United Kingdom [33]. Although the questionnaire consists of three scales, only the parent alliance scale (4 items), rated on 4-point Likert scales ranging from *most of the time* to *almost never* with a Cronbach’s alpha of .69, was used for this study [2].

#### Parent-team alliance from parents’ perspective

The empathy and understanding questionnaire (EUQ) is a questionnaire aimed at assessing the parents’ perspective on the therapeutic alliance with team members in a child (semi) residential psychiatric setting [34]. Elvins and Green [1] report the initial psychometric properties of the EUQ as adequate. After permission from the original author, the EUQ was translated and its psychometric qualities were investigated in the Netherlands in accordance with the guidelines of van Widenfelt and colleagues [35]. Independent translation (by three psychologists) and back translation (by two native speakers) of the items and response categories were conducted and consensus was reached in brainstorming sessions. A subsequent explorative factor analysis for mothers (*N* = 67) and fathers (*N* = 50) revealed unifactorial solutions. The Dutch questionnaire consists of five items with ready-made answer categories. Cronbach’s alpha for both mothers’ and fathers’ reports of the EUQ were acceptable (mothers, .77 and fathers, .79). The final back-translated version of the EUQ is presented in Additional file 1: Appendix.

#### Child’s strengths and difficulties

The Dutch version of the strength and difficulties questionnaire (SDQ) is a 25-item measure [36] assessing both the child’s strengths and difficulties. The questionnaire has five subscales in addition to a total score: emotional problems (EMO), conduct problems (COND), hyperactivity (HYP), peer problems (PEER), and prosocial behaviour (PROSO). There are three response categories, ranging from ‘not true’ (0) to ‘certainly true’ (2). The sum of scales 1–4 results in a total difficulty score with a minimum of 0 and a maximum of 40. In contrast to the other scales, a high score on the prosocial scale indicates strengths. Cronbach’s alpha was .82 for the parent version of the total score and between .57 and .85 for the subscales [36]. Cronbach’s alpha was .87 for the teacher version of the total score and ranged between .70 and .88 for the subscales [36].

### Procedures

The research plan, which was part of a larger study, has been approved by the Medical Ethical Committee of the Leiden University Medical Center. The research was judged as falling outside of the WMO (Dutch Medical Research in Human Subjects Act) as data was collected to improve treatment, which made written consent unnecessary. All participants referred to the semi-residential treatment were informed before the first contact that research was an integrated part of their treatment. Informed consent was subsequently obtained from participants of the 46 children during the admission process to the semi-residential setting. Only one referred client was not included in the study as parents lacked a sufficient command of the Dutch language. Patient data were managed in line with Dutch ethical guidelines, that is, the Personal Data Protection WGBO (Agreement on Medical Treatment Act) and WBP (Personal Data Protection Act).

For the present study, longitudinal assessments of the SDQ, EUQ and FEQ were used. The first SDQ assessment was before the intake; the first EUQ/FEQ assessment occurred after 6 weeks of treatment. Subsequent assessments were planned with 3-months intervals as long as treatment continued. Information on sociodemographics (e.g., education level of parents) and DSM-IV (diagnostic and statistical manual of mental disorders) classifications (DAWBA: development and well-being assessment) was collected as part of standard procedures during the client’s admission for the semi-residential psychiatric unit [36].

### Statistical analyses

The maximum of missing values for a given scale for the EUQ and FEQ was no more than one missing item. In case of one missing item per scale, these were replaced by using the person mean substitution method [37]. Descriptive statistics were conducted with SPSS (version 20.0).

The development of the alliance and outcome variables was analyzed with multilevel modeling carried out with MLwiN (version 2.22) [38]. The assessment times (first level) were nested within the individuals (second level), so dependencies between assessment times for the same child were accounted for. The advantage of using multilevel analysis with repeated measures is that all available data could be incorporated into the analysis, including data from participants that missed one or more measurement occasions. Group assignments were entered into the equation as an independent variable to assess average treatment effects over time. In addition, to assess treatment effects on alliance at the different time points the alliance variable assessment time (represented by dummy variables) and the interaction between time and group allocation was added to the model. All analyses on alliance were adjusted for location and education level; all analyses on strengths and difficulties of the child were adjusted for location and age of the child.

## Results

### Attrition analysis

No significant differences in completion rates for the EUQ were found between the locations (*p* = .20) and the treatment conditions (*p* = .41). Also for the SDQ’ reports no difference was found between locations (caregiver: *p* = .52; group worker *p* = .15) and treatment conditions (caregiver: *p* = .21; group worker: *p* = .06). However, for the FEQ there was a significant difference in completed questionnaires between the two treatment locations (*p* = .01). For treatment location 2, completion rates ranged between 30 and 65 %, which excluded this data when analyzing the FEQ. The licensed clinical psychologist mentioned time pressure as the main reason. The number of days between assessment times was variable (EUQ: *M* = 84, *SD* = 25; FEQ: *M* = 86, *SD* = 24; SDQ caregiver: *M* = 89, *SD* = 42; SDQ group worker: *M* = 103, *SD* = 52), however, not different between the comparison and experimental group (EUQ: *p* = .10; FEQ: *p* = .67; SDQ caregiver: *p* = .278; SDQ group worker: *p* = .46).

### Results integrity procedure

Of the 46 clients, 18 evaluation meetings were taped on DVD, 13 from location 1 and 5 from location 2. The main reason for not taping evaluation meetings was the failure to set up the camera. The first rater assessed all the DVDs on treatment integrity. The mean score per aspect on all DVDs was (1) emphasizing partnership, 2.4 (*SD* = .92); (2) agreement on a shared explanatory model of illness, 2.8 (*SD* = .61); (3) agreement on goals, 2.9 (*SD* = .68); (4) agreement on tasks, 2.6 (*SD* = .85); (5) emphasizing the effect of treatment, 2.8 (*SD* = .55); (6) zooming in on strengths of child and parents, 2.8 (*SD* = .79); (7) enhancing parents’ reflective state, 2.6 (*SD* = .62), and (8) parents overall satisfaction with treatment, 3.3 (*SD* = .59). The intraclass correlation coefficient between the coder and the reliability coder was .54 (*p* = .00).

### Pre-intervention equivalence of groups

As can be seen in Table 1, the primary classification of the children varied significantly (*p* = .04) between the comparison and experimental group with slightly more behavior disorders in the experimental group and slightly more anxiety disorders in the comparison group. Furthermore, children in the experimental group (248 days) attended day treatment for significantly (*p* = .04) fewer days than the comparison group (328 days). For the other baseline characteristics, no significant group differences were found in the scores from the pre-test (*p* = .24 to .84).

### Descriptive statistics of participants for each assessment

The alliance scores per group over five assessments for the primary caregivers on the EUQ and one case manager on the FEQ are shown in Table 2. A higher score reflects stronger alliances. Caregivers’ alliance scores for the comparison group ranged from 14.4 to 14.9, while in the experimental group from 15.2 to 17. Case manager’ alliance scores ranged from 10.5 to 14.7 in the comparison group and from 13.3 to 16 in the experimental group.

**Table 2:** Means (SD) of alliance scores across assessments of parents on the EUQ and of clinical psychologist on FEQ

*T*	EUQ comparison			EUQ experimental		
	*N*	%	Total score	*N*	%	Total score
1 (6–8 weeks)	19	86	14.7 (2.1)	17	71	15.2 (1.6)
2 (3–4 months)	21	95	14.9 (1.0)	21	91	15.6 (1.7)
3 (6–7 months)	16	76	14.9 (1.8)	18	95	16.1 (1.4)
4 (9–10 months)	9	69	14.4 (1.0)	11	73	16.3 (1.6)
5 (12–13 months)	9	90	14.7 (1.1)	3	38	17.0 (1.7)

	FEQ comparison			FEQ experimental		
	*N*	%	Parent score	*N*	%	Parent score
1 (6–8 weeeks)	0	0	–	14	80	13.3 (1.8)
2 (3–4 months)	8	80	10.5 (1.9)	16	100	14.3 (1.3)
3 (6–7 months)	9	90	12.2 (2.9)	13	100	15.0 (1.5)
4 (9–10 months)	8	100	13.1 (2.4)	9	82	15.0 (1.5)
5 (12–13 months)	6	100	14.7 (1.8)	3	100	16.0

Values given are means (*SD*); % = Percentage of completed questionnaires; higher scores reflected stronger alliances

In Table 3 the strength and difficulties scores of caregivers’ and group workers’ are presented per group over the five assessments. Externalizing symptoms in particular decreased over time. Caregivers’ hyperactivity scores decreased from 7.3 to 6.9 in the comparison group versus 7.4 to 5.5 in the experimental group and conduct symptoms from 3.8 to 3.1 in the comparison group versus 4.5 to 2.7 in the experimental group. For group workers, hyperactivity symptoms scores decreased from 7.1 to 5.5 in the comparison group and 6.2 to 4.2 in the experimental group and conduct symptoms scores from 3.5 to 3.9 in the comparison group and decreasing from 3.4 to 2.2 in the experimental group.

**Table 3:** Means (SD) of strength and difficulties scores across assessments of parents and group workers on the SDQ

*T*	SDQ parents comparison group					SDQ parents experimental group				
	Emo	Cond	Hyp	Peer	Proso	Emo	Cond	Hyp	Peer	Proso
1	5.0 (2.7)	3.8 (2.3)	7.3 (2.6)	4.6 (1.9)	6.5 (2.4)	6.8 (3.0)	4.5 (2.3)	7.4 (3.0)	4.5 (2.2)	5.7 (2.4)
2	5.6 (2.4)	3.5 (2.8)	7.1 (2.3)	4.3 (2.7)	6.3 (2.0)	6.5 (2.6)	3.6 (1.3)	6.0 (2.2)	4.9 (2.3)	6.5 (2.4)
3	5.2 (2.4)	3.3 (2.6)	7.6 (2.7)	4.4 (2.3)	6.4 (2.5)	5.3 (2.6)	3.4 (2.5)	6.3 (2.8)	4.0 (2.3)	6.3 (2.4)
4	5.3 (2.3)	3.7 (2.5)	7.1 (2.8)	4.7 (1.8)	6.0 (2.1)	5.0 (2.7)	2.8 (1.9)	5.7 (2.9)	3.7 (2.5)	6.3 (2.4)
5	4.3 (2.1)	3.1 (1.9)	6.9 (2.1)	4.8 (2.3)	6.6 (1.8)	4.7 (2.7)	2.7 (1.8)	5.5 (2.9)	4.1 (2.1)	5.9 (2.6)

*T*	SDQ group workers comparison group					SDQ group workers experimental group				
	Emo	Cond	Hyp	Peer	Proso	Emo	Cond	Hyp	Peer	Proso
1	6.0 (2.0)	3.5 (2.3)	7.1 (2.8)	4.6 (2.3)	3.9 (2.3)	5.0 (3.1)	3.4 (2.7)	6.2 (3.5)	4.4 (2.4)	4.3 (2.2)
2	5.7 (2.4)	3.6 (3.2)	5.4 (4.0)	4.1 (2.6)	4.1 (2.7)	5.9 (2.4)	2.9 (2.3)	4.5 (3.0)	4.7 (1.9)	4.3 (2.4)
3	5.4 (2.2)	4.2 (3.0)	4.8 (3.4)	4.0 (2.7)	4.2 (2.9)	6.8 (2.8)	2.8 (2.6)	4.4 (3.6)	4.7 (2.5)	3.9 (2.0)
4	5.2 (1.9)	3.0 (2.6)	5.5 (3.4)	3.6 (2.9)	4.5 (3.2)	5.5 (2.8)	2.2 (1.7)	4.5 (2.8)	3.5 (2.1)	4.5 (1.9)
5	4.5 (2.8)	3.9 (3.1)	5.5 (3.8)	4.3 (2.3)	3.9 (2.5)	5.4 (2.9)	2.2 (2.3)	4.2 (3.8)	4.6 (2.1)	4.3 (1.0)

Values given are means (*SD*). T1 = Before intake, T2 = 3–4 months, T3 = 6–7 months, T4 = 9–10 months; T5 = 12–13 months; Comparison parents n = 19, 19, 19, 15 and 10; Experimental parents: n = 18, 10, 21, 18 and 13; Comparison Group workers: n = 14, 19, 20, 16, 8; Experimental group workers: n = 19, 24, 19, 15, 9; Higher scores reflected more symptoms (except for the Prosocial Scale)

### Intervention effects

Multilevel analyses (see Table 4) showed that the alliance scores of the primary caregivers were significantly higher in the experimental group compared to the comparison group (EUQ: *β* = .89; *SE* = .33; *p* = .01). Also for the case manager’ reports, there was a significant group effect on the parent alliance scales (FEQ: *β* = 1.94; *SE* = .56; *p* = .00). Next, when examining the development of the therapeutic alliance between the groups between assessment times, for the EUQ as well as for the FEQ, no significant interaction effects were found.

**Table 4:** Multilevel analyses of intervention effect on parent-team alliance, alliance over time and strengths and difficulties child

		EUQ caregivers report^a^				FEQ case manager report		
		Δ	*B (SE)*	*p*		Δ	*B (SE)*	*p*
*Alliance*			.89 (.33)	.01*			1.94 (.56)	.00**
Group*time T1–2	C	.27	−.01 (.33)	.98	C	–	–	–
	I	.44			I	.96		
Group*time ΔT2–3	C	−.08	−.26 (.37)	.48	C	1.72	−1.08 (.92)	.24
	I	.49			I	.75		
Group*time ΔT3–4	C	−.43	−.21 (.45)	.64	C	.90	−.94 (.98)	.34
	I	.16			I	−.22		
Group*time ΔT4–5	C	.33	.27 (.53)	.61	C	1.54	.44 (1.35)	.74
	I	.73			I	1.22		

Strength and difficulties		SDQ caregiver report^a, b^				SDQ group worker report^a, b^		
			*B (SE)*	*p*			*B (SE)*	*p*
Emotional problems			.59 (.73)	.42			.27 (.59)	.65
Conduct problems			.40 (.56)	.48			−.78 (.71)	.27
Hyperactivity			−1.38 (.55)	.01*			−.63 (.82)	.37
Peer problems			−.06 (.63)	.92			.64 (.60)	.29
Prosocial behaviour			−.88 (.62)	.16			.07 (.56)	.90

Values given are *B* estimates (*SE* standard error), except for Δ = Difference of the mean scores between two assessment times

*C* comparison group; *I* intervention group; *T1* 6–8 weeks; *T2* 3–4 months; *T3* 6–7 months; *T4* 9–10 months; *T5* 12–13 months

* *p* < .05, ** *p* < .01

^a^Adjusted for location

^b^Adjusted for age child, time of admission and a behavior disorder on AXIS I

As a result of the difference between the two groups, the multilevel analyses on the SDQ were additionally adjusted for treatment length and a behavior disorder classification. As can be seen in the lower part of Table 4, most multilevel analyses with SDQ’ reports did not result in significant changes in symptoms over the course of treatment on the different subscales. The only exception was a significant decrease of hyperactivity problems in the experimental group compared to the comparison group (SDQ, hyperactivity scale:* β* = −1.38; *SE* = .55; *p* = .01) according to caregivers’ reports.

## Discussion

A growing body of research emphasizes the parent alliance as a crucial concept in treatment effectiveness for children. Especially in a semi-residential setting, investment in a strong therapeutic alliance with parents is valued by clinicians and is seen as an important factor to improve treatment. However, to our knowledge, there are no scientific guidelines for treatment team members to learn how to strengthen parent-team alliances. For this purpose, we derived parent-team alliance-building strategies from the literature and did a first attempt to investigate their effectiveness in a semi-residential psychiatric setting. The main finding from this study is that structured investment of treatment team members in the parent-team alliance in children’s semi-residential treatment was effective in enhancing the strength of this alliance. Longitudinal assessments of both the caregivers’ and the clinical psychologist’ perspectives showed this effect. The developmental pattern of the strength of the alliance did not differ between treatment conditions. In addition, a significant decrease was found of child’s hyperactive behavior in the experimental group, yet, no such decrease was found on the other symptom scales. In child (semi) residential literature, qualitative published studies emphasize the importance of strengthening the parent-team alliance; now, this is additionally supported by preliminary quantitative results from the current study.

Primary caregivers as well as the clinical psychologist value the strength of the parent-team alliance significantly stronger after team members’ investment in alliance-building strategies. Building an alliance with parents in a (semi) residential setting can be quite challenging, due to possible feelings of tension and ambivalence that are likely inherent to this treatment [20, 21]. Apparently, alliance-building strategies found to effectively strengthen therapeutic alliance in other treatment settings also effectively strengthen the parent-team alliance in a semi-residential setting. Our results showed that a whole multidisciplinary team can be trained, instead of only one therapist, as has been done in earlier studies [14–17]. Semi-residential treatment is a complex package of treatment interventions, which differs for each client involved. Investment in a common process factor, like the therapeutic alliance, which is essential for each client, is therefore a valuable effort. The effective alliance building strategies include partnership [27], positive attributes [30] and explicit evaluation of the alliance [31]. If parents feel the treatment team is listening to them, they may be more incited to participate in their child’s treatment and may feel more responsible for the actual treatment result.

Unique of this study is that the development of the parent-team alliance was longitudinally evaluated during treatment. The pattern of the development of the therapeutic alliance was no different when comparing the comparison group and the experimental group. Apparently, the alliance scores are just overall higher for the experimental group than for the comparison group and increase both gradually. In retrospect, the alliance strategies are already intensively in effect before the child starts semi-residential treatment, so it is not surprising a difference was found from the beginning of treatment. Thus, strong alliance building with parents is essential from the beginning of treatment.

A stronger parent alliance has been associated with better treatment outcomes in children’s residential treatment; therefore, strengthening parent alliance may improve effectivity of children’s semi-residential treatment. McLeod’s [11] meta-analysis showed that the effect size of the alliance-outcome association in outpatient treatment was practically identical for the youth alliance and the parent alliance, indicating both relationships play a crucial role for improving treatments. Caregiver reported hyperactivity problems decreased significantly in our experimental group. In addition, although not significant, group workers reports of conduct symptoms in the experimental group were lower than in the comparison group. There was no effect on the internalising symptoms, peer problems or prosocial behaviour of the child. However, as the parent alliance has been repeatedly and mostly associated in the literature with a decrease of externalising symptoms [7, 39], it is promising that strengthening parent alliance leads to an improvement of the child’s hyperactive symptoms according to caregivers.

This exploratory systemic evaluation, done in the complex setting of semi-residential psychiatry, has some limitations, which requires cautious interpretation of results. Firstly, a randomized controlled trial would have been preferred to evaluate the direct effectiveness of an important treatment factor. However, the severity of patients’ disorders and their often urgent need for hospitalization made randomizing into groups both practically and ethically difficult. The chosen A–B design with repeated measuring and different reporters strengthened the design, warranting notwithstanding tentative conclusions of significant changes for this institute. Secondly, only the clinical psychologist from the treatment team, who was also the one implementing the alliance strategies, reported on the parent alliance, so one cannot assume those alliance ratings were fully independent or not biased. Clinical psychologists are often more skilled in common relational skills and are more integrative in their treatment orientation, which results most probably in a better utilization of good alliances for treatment success [40, 41]. The increased strength of the alliance in the experimental group may be the result of an enthusiastic attitude towards the alliance strengthening strategies. Thirdly, although a high number of children with a classification of PDD is quite common in semi-residential treatment in The Netherlands [42], this should be taken into account when generalizing these findings to other semi-residential settings. Finally, some factors complicated the treatment integrity procedures like (a) no specific treatment was evaluated; but therapeutic strategies added to (semi) residential treatment of children and (b) the adherence and competence of not *one* therapist but that of a whole treatment team was assessed. Maybe, as a result, a relatively low interrater agreement score was reached. However, given the generally low rate of incorporating treatment integrity assessment in effectiveness research; the current effort to implement integrity procedures is a strength of this study [43, 44].

To confirm our conclusions regarding the parent-team alliance as an important common process factor in semi-residential psychiatry, it is recommended to perform a multi-center research with more (semi-) residential units with differentiated psychopathology and more treatment teams. This way, comparisons can be made between semi-residential and residential treatment, between treatment teams, between different groups of psychopathology and between age groups. Additionally, randomization on unit level is recommended to examine more thorough the effectiveness of the alliance strengthening strategies on child’s symptoms. Furthermore, the development of the parent-team alliance is likely to be interconnected with the development of the child-team alliance. Thus, ideally, future alliance studies include the child and parent alliance simultaneously. Alliance building strategies could be developed for the child-parent-team alliance, instead of only for the parent-team alliance.

## Conclusions

In the youth alliance literature, it remains relatively unknown how the parent alliance could be effectively strengthened. This is the first study that contributes to the development of clinical practices for clinicians to strengthen the parent alliance. Parents of a child with complex psychiatric disorders deserve intensively structured attention from treatment team members during their child’s semi-residential treatment.

## Additional file

10.1186/s13034-016-0108-5 Appendix.

## Abbreviations

WGBO: Agreement on Medical Treatment Act
WBP: Personal Data Protection Act
DAWBA: development and well-being assessment
SDQ: strengths and difficulties questionnaire
FEQ: family engagement questionnaire
EUQ: empathy and understanding questionnaire
DSM-IV: diagnostic and statistical manual of mental disorders
PDD: pervasive development disorder
ADHD: attention deficit hyperactivity disorder
ODD: oppositional defiant disorder
