# Supplementary material for: Longitudinal results of strengthening the parent-team alliance in child semi-residential psychiatry: does team investment make a difference?
Source: Child Adolesc Psychiatry Ment Health. 2016 Jul 18;10:22. doi: 10.1186/s13034-016-0108-5 (PMC4948094; doi:10.1186/s13034-016-0108-5)
Supplement: Supplementary file 1 — 10.1186/s13034-016-0108-5 Appendix. [file 13034_2016_108_MOESM1_ESM.doc]

**Appendix**

**Empathy and understanding questionnaire**

For all the questions there are four possible response categories. You can check off the answer in the little box that fits best with what you think. Thank you for your cooperation and time.

**What effect do you think that the treatment program will have on your child?**

I think that all the problems that we experience will be completely solved.

I think that it will help deal with many of the problems that we experience.

I don’t think that it will help much in solving the problems that we experience.

I think that it is a complete waste of our time and that it won’t help.

**Do you think that the treatment staff/clinicians listen well to what you have to say during the treatment sessions?**

They have already decided what they are going to do and don’t listen at all.

They try to listen to our opinion, but don’t spend much time on it or pay attention to it.

They recognize the importance of our opinion and listen to it.

The entire treatment is adapted to or has taken our ideas into consideration and the clinician always listens attentively to it.

**Do you think that the treatment team really understands what your family problems are and how difficult it is to deal with them?**

No, they have no idea what it is like for us and they are not in a position to understand what problems we are dealing with.

I think that they understand some problems but they don’t understand how difficult it is for us to deal with the problems.

I think that they have a correct idea or understanding of the problems we are dealing with and that they understand most of our problems.

Yes, I think that they understand exactly what our problems are just as well or even better than we can.

**How do you think that the treatment team views you and your child?**

I think that they see us as just another “file” number. They don’t really care about us and don’t try to get to know us as people.

I think that they try a little to get to know us so the therapy may go smoother, but I don’t think that they really care about us as people.

I think that they are friendly towards us as people. I think that they care about all their clients.

I think that the team has built a close bond with us. I think that they do all they can to help us.

**How important and involved are you in the treatment program that is given to your child?**

I think that I am the most important person. The clinicians are there, for the most part, to help me work together with my family.

My entire family is important in the collaboration with the team. I am committed to really help and do my part.

I am, for the most part, told what I should do by the clinician. I am only asked to participate at certain moments and my ideas and opinions aren’t that important.

The team carries out the entire treatment. I am not that involved and my ideas are not that important.
